# Convergent validity of 12-item World Health Organization Disability Assessment Schedule (WHODAS 2.0) among people with neck pain

**DOI:** 10.1371/journal.pone.0315676

**Published:** 2025-03-11

**Authors:** Aleksandra Karklins, Guna Be¯rzin¸a, Mikhail Saltychev

**Affiliations:** 1 Finnish Student Health Services, Turku, Finland; 2 Department of Rehabilitation, Riga East University Hospital, Riga, Latvia; 3 Department of Physical and Rehabilitation Medicine, Turku University Hospital and University of Turku, Turku, Finland; BRAC University, BANGLADESH

## Abstract

**Objective:**

To explore the convergent validity of 12-item World Health Organization Disability Assessment Schedule (WHODAS 2.0) comparing it to Neck Disability Index (NDI).

**Design:**

Cross-sectional cohort study.

**Subjects/Patients:**

962 patients visiting a university outpatient Physical and Rehabilitation Medicine Clinic due to musculoskeletal complaints.

**Methods:**

Spearman´s rank correlation between WHODAS 2.0 and NDI.

**Results:**

The average age was 49.2 (SD 14.5) years, 67% were women. Of all the possible 143 correlations between WHODAS 2.0 and NDI, 99 (69%) were positive, significant and strong or, at least, moderate. The correlation between the composite scores of two scales was strong. The weakest correlations were seen for the NDI items ‘pain intensity and ‘headaches’.

**Conclusion:**

Most of the items and the composite scores of the WHODAS and the NDI demonstrated significant positive correlations. Pain intensity, as defined by the NDI, did not correlate with disability severity measured by the WHODAS 2.0. Also, the NDI items ‘headaches’ and ‘sleeping’ were associated with the WHODAS 2.0 only loosely. It seems that one of these two scales may not directly be substituted by another. When used simultaneously, The WHODAS and the NDI may complement each other covering comprehensively the different dimensions of functioning among people with neck pain.

## Introduction

The World Health Organization Disability Assessment Schedule (WHODAS 2.0) has been introduced as a generic scale to measure disability among adults across all cultures and settings regardless of the specific cause of disability [1]. The WHODAS 2.0 has been found to be a reliable and valid tool to assess disability in patients with musculoskeletal pain [2,3]. It has demonstrated excellent psychometric properties including factor structure, test-retest reliability, high internal consistency and good discrimination ability [4]. The WHODAS 2.0 has been found to be well associated with the WHO minimal generic data set and different patient-reported outcome measures (PROMs), such as the Bath Ankylosing Spondylitis Functional Index (BASFI), the Dougados Functional Index (DFI), the SF-36, a pain numeric rating scale (NRS) and the EQ-5D-5L among others [5–10]. The WHODAS 2.0 has quickly become a familiar easy-to-use scale among both researchers and clinicians working with very different health disorders. In turn, the Neck Disability Index (NDI) is a disease-specific tool for measuring disability in patients with neck pain [11–13]. Through the last three decades, the properties of the NDI have extensively been studied confirming its high test-retest reliability and internal consistency and good construct validity [12].

As the WHODAS 2.0 is a relatively new scale, its convergent validity should be confirmed comparing it with other PROMs across different populations and settings. By assessing the convergent validity of a scale, one may assure that the scores obtained from a newer scale are in line with older scales measuring similar constructs. Additionally, if two scales – a newer and an older one – are quite similar, then the question arises – could it be possible to substitute one by another avoiding redundant composite tests. If, on the other hand, two scales assess different domains of a same construct, then should they be used together complementing each other?

Although the NDI and the WHODAS 2.0 are widely used among pain patients, the joint use of these two measures in the same research setting is either rare or completely absent. However, it can be assumed that these situations will occur more frequently as the WHODAS 2.0 becomes more common. The NDI and WHODAS 2.0 measure the same area of functional ability that the ICF classification calls performance - the person’s functioning in their usual environment. However, they largely cover different dimensions of this concept – the NDI is more physically focused while the WHODAS 2.0 measures much more broadly the person’s participation limitations. Because of these differences, the hypothesis of this study was that although some items of the measures correlate well, others show only weak correlations, if at all.

The objective of this study was to explore the convergent validity of the WHODAS 2.0 compared to the NDI. The results may help to choose the right set of scales for a particular situation. If the association between the two scales will appear to be close to perfect, then in some situations, the disease-specific NDI may possibly be substituted by the generic WHODAS 2.0.

## Methods

This was a cross-sectional cohort study of consecutive patients who had visited a university outpatient clinic of Physical and Rehabilitation Medicine between 2014 and 2017 [14,15]. The survey included the 12-item self-administered WHODAS 2.0, the NDI, and questions on demographics and pain intensity. The survey was sent in a paper-version to all the patients visiting the clinic. The patients filled out the survey before they visited a clinic. Of 3,150 patients, 1,988 responded to a questionnaire (response rate 63%). Of 1,988 patients, 962 responded to both the WHODAS 2.0 and the NDI and, were included in this study. The diagnoses were gathered from electronic patient records. The study protocol was approved by the Ethics committee of Hospital District of Southwest Finland (ETMK 60/180/2012).

The self-administered 12-item WHODAS 2.0 contains 12 items, covering the most common limitations of functioning presented during the last 30 days. A Likert-like five-level ordinal scale is used to define the severity of limitation with ‘zero’ denoting ‘no limitation’ and ‘four’ denoting ‘extreme limitation or inability to function’. A simple scoring approach was used and a total score was the sum of all item scores [1]. The score of 0 points denoted the best possible functioning and total independence, while the score of 48 points denoted the worst possible disability and total dependence [4,15].

The NDI includes 10 items covering activities of daily living, pain, and concentration. A Likert-like six-level ordinal scale was used to define the severity of disability caused by neck pain with ‘zero’ denoting ‘no pain or disability’ and ‘five’ denoting ‘most severe pain or disability’. The total score was expressed in points and calculated as [(sum of all item scores)/(number of items with non-missing responses *  5)] * 100. The higher the NDI score, the worse disability perceived by a respondent.

Pain was measured in points of numeric rating scale from 0 (‘no pain’) to 10 (‘worst possible pain’) during the last 30 days. Age was defined in full years at the time of response to a questionnaire. Body mass index (BMI) was self-reported, calculated as body weight divided by squared height and expressed in kg/m^2^. Sex was defined as biological sex men vs. women. The respondents were asked concerning their marital status as ‘living alone’ vs. ‘living with family’ and the responses were reported as ‘single’ vs. ‘co-habiting’. Smoking status was understood as current smoking at the time of response and dichotomized as ‘yes’ vs. ‘no’

### Statistical analysis

All the descriptive statistics were presented as absolute numbers and percentage or as means and standard deviations (SDs), when appropriate. Correlations between the WHODAS 2.0 and the NDI were assessed by using a Spearman’s rank correlation coefficient: 0.40-0.69 was considered ‘strong’; 0.30-0.39 ‘moderate’; 0.20-0.29 ‘weak’; and < 0.20 ‘none or negligible correlation’ [16]. Two-tailed p-values were reported considering values < 0.05 to be statistically significant. There are no commonly recommended thresholds for the strength of correlation. In this study, recommendations by Quinnipiac University (USA) were used as they were considered having more levels to describe the strength of correlations (very strong – strong – moderate – weak – negligible) than, e.g., a gradation system by Dancy & Reidy, used mostly in psychology (strong – moderate – weak) [17]. A sensitivity analysis was performed using the gradation system by Dancy & Reidy and the results were presented as a supplementary table (S1 Table).

Considering the uneven sex distribution, a sensitivity test was applied calculating correlation coefficients for men and women (S2 Table).

Of the 962 observations, there were 436 incomplete responses to the WHODAS 2.0 and 348 to the NDI. Considering that the main objective of the study was to assess the correlations between individual items, all the available data were used, including complete and incomplete responses. All the demographic data were available for all the respondents

All the analyses were conducted using Stata/IC Statistical Software: Release 17, College Station (StataCorp LP, TX, USA).

## Results

**Fig 1:**
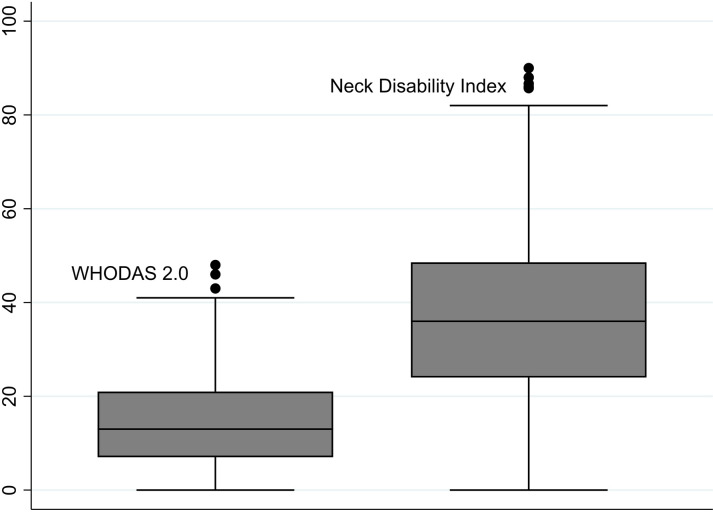
Box plots – distributions of composite scores of WHODAS 2.0 and NDI.

The mean age of 962 patients was 49.2 (SD 14.5) years. Of them, 647 (67%) were women (Table 1). The average BMI was 26.8 (SD 6.5) kg/m^2^. Of the patients, 669 (74%) were co-habiting and 237 (26%) were smoking. The mean severity of ‘steady’ pain was 6.5 (SD 1.9) points. Respectively the ‘worst’ pain severity was 8.2 (SD 1.6) points. The mean WHODAS 2.0 total score was 14.4 (SD 9.6) points (Fig 1). The mean NDI total score was 37.5% (SD 17.5%). In total, 828 (86%) patients had main diagnoses of ‘M’ domain of the International Classification of Diseases, 10^th^ Edition, with the most frequent diagnosis M54 ‘Dorsalgia’ in 34%.

**Table 1:** Descriptive characteristics of the sample.

Demographics, pain severity, NDI ^a^ score, WHODAS 2.0 ^b^ score	Mean	Standard deviation
Age, years	49.2	14.5
Body mass index, kg/m^2^	26.8	6.5
Pain intensity, points		
Average	6.5	1.9
Worst	8.2	1.6
NDI total score, points	37.4	17.5
WHODAS 2.0 total score, points	14.4	9.6
Demographics	N	%
Gender	962	
Men	315	32.7
Women	647	67.3
Marital status	901	
Single	232	25.8
Co-habiting	669	74.3
Smoking	915	
Smoker	237	25.9
Non-smoker	678	74.1
Main diagnoses ^c^	N	%
M Diseases of the musculoskeletal system and connective tissue	828	86
M54 Dorsalgia	323	34
M79 Other and unspecified soft tissue disorders	60	6
M50 Cervical disc disorders	45	5

^a^Neck Disability Index;

^b^World Health Organization Disability Assessment Schedule 2.0 (12-item);

^c^International Classification of Diseases, 10^th^ Edition

Of all the possible 143 correlations, 45 (32%) were moderate and 44 (31%) strong (Table 2). The lowest Spearman´s rank correlations between the WHODAS 2.0 total score and the NDI items were observed for ‘pain intensity’ 0.25, ‘headaches’ 0.27 and ‘sleeping’ 0.39. The associations between items with similar expressions were high: correlation between ‘concentration’ and ‘concentrating’ was 0.64 and between ‘work’ and ‘work or school’ 0.66. All the correlation coefficients were positive and significant with *p*-values < 0.0001.

**Table 2:** Spearman´s rank correlations between the 12-item WHO Disability Assessment Schedule 2.0 and the Neck Disability Index. All the p-values < 0.0001.

Neck Disability Index items	WHODAS 2.0 items												
	Standing	Household care	Learning new task	Community activities	Emotional affection	Concentrating	Walking	Washing	Dressing	Dealing with strangers	Maintaining friendship	Work or school	Total
Pain intensity	0.16	0.23	0.13	0.21	0.19	0.21	0.17	0.21	0.16	0.13	0.18	0.25	0.25
Personal care	0.32	0.42	0.2	0.36	0.25	0.29	0.35	0.56	0.53	0.18	0.23	0.35	0.47
Lifting	0.38	0.48	0.26	0.41	0.26	0.28	0.41	0.41	0.32	0.21	0.29	0.45	0.49
Reading	0.32	0.36	0.25	0.36	0.27	0.34	0.31	0.3	0.23	0.25	0.3	0.36	0.42
Headaches	0.19	0.23	0.11	0.29	0.21	0.24	0.17	0.18	0.15	0.17	0.23	0.24	0.27
Concentration	0.39	0.38	0.46	0.49	0.47	0.64	0.38	0.37	0.25	0.43	0.49	0.41	0.59
Work	0.42	0.55	0.3	0.48	0.34	0.41	0.46	0.48	0.38	0.28	0.35	0.66	0.61
Driving	0.34	0.4	0.27	0.43	0.32	0.37	0.33	0.37	0.27	0.28	0.32	0.43	0.48
Sleeping	0.28	0.33	0.22	0.29	0.28	0.32	0.31	0.35	0.3	0.25	0.24	0.33	0.39
Recreation	0.35	0.43	0.26	0.46	0.34	0.34	0.37	0.35	0.28	0.21	0.32	0.45	0.49
Total	0.43	0.52	0.34	0.51	0.39	0.47	0.45	0.49	0.38	0.34	0.4	0.55	0.6

All the p-values < 0.0001

0.01 to 0.19 negligible correlation

0.20 to 0.29 weak correlation

0.30 to 0.39 moderate correlation

0.40 to 0.69 strong correlation

When applying a correlation gradation by Dancy & Reidy, of all the possible 143 correlations, 41 (29%) were moderate and the rest were weak (S1 Table). While the strength of correlations according to this gradation was considered overall weaker than one used as a main assessment approach, the results otherwise showed similar trends. When calculating correlation coefficients separately for men and women, despite some differences the correlation matrices were, however, alike for both sexes (S2 Table).

## Discussion

In this cross-sectional study of 962 patients with neck pain, moderate or strong, positive, and significant correlations were observed in about two thirds of all the possible associations between the WHODAS 2.0 and the NDI. Respectively, another one third of all the possible associations were weak or negligible. Two NDI items ‘pain intensity and ‘headaches’ had only negligible or weak correlations with the items of the WHODAS 2.0. The NDI item ‘sleeping’ had weak to moderate correlation with any of the WHODAS 2.0 items. Similarly expressed items were strongly correlated, e.g., ‘concentration’ vs. ‘concentrating’, or ‘work’ vs. ‘work or school’. The correlations between the total scores of the two studied scales were strong.

The generalization of the results might be weakened by the demographics of the study sample – a particular age group around 50 years predominated by women. The patients were visiting a highly specialized university clinic and therefore, the results might not be applicable to the entire population of people with neck pain or to primary care patients with neck problems. It should also be considered that some of the patients were visiting the clinic for another main reason than neck pain. Some of the items presented in both the WHODAS 2.0 and the NDI are very similar. For example, “personal care” (NDI) vs. “washing” and “dressing” (WHODAS 2.0) or “work” vs “work or school”. Answering to similar questions during the same survey might influence the psychometrics of responses. The results were limited to the self-administered short 12-item WHODAS 2.0, and they might be different for other versions of the WHODAS 2.0.

While no previous studies assessing the direct interrelations of the WHODAS 2.0 and the NDI could be identified, the present results could, however, be indirectly compared with some previous research. Similarly to the present findings, strong correlations between the WHODAS 2.0 and almost 20 other scales have been reported earlier [18]. Interestingly, the WHODAS 2.0 has previously demonstrated a weaker association with the Barthel index [18]. This observation may reflect the difference between concepts of capacity (what a person is able to do in the best-arranged conditions) and performance (how well a person is performing in their usual environment). While the WHODAS 2.0 exclusively measures performance, Barthel index is a measure of capacity. Taking that into consideration, the strong correlations between the WHODAS 2.0 and the NDI was expected as both scales are the measures of capacity with partially overlapping questions. Previous research has also noticed that the WHODAS 2.0 might show different strength of correlations for physical and mental domains of functioning when comparing with other scales. For example, the WHODAS 2.0 has demonstrated strong correlation with the physical component score of SF-36 but only moderate with its mental component score [5,10].

The composite scores of questionnaires are without a doubt of great importance in research. They are useful when evaluating the prevalence or incidence of an event, or when comparing, e.g., changes in disability severity across a large sample or an entire population. However, a composite score says little when describing severity of disability of a particular person. It is self-evident that the individual items of the WHODAS 2.0 or the NDI may contribute differently to their composite scores. For example, the composite score may mostly come from the “physical” domain of the WHODAS 2.0, or it may exclusively be the sum of “psychosocial” items scores, resulting in similar summary scores in both cases, which are not, however, comparable. Thus, when describing the functioning level of an individual, it would be more informative (and more in the spirit of the ICF classification) to describe disability severity for each individual item, resulting in a functional profile rather than in a single number. However, only a few studies have assessed the associations between the individual items of the WHODAS 2.0 and the individual items of other scales.

Probably, the most comparable with the present results are the results of studies comparing the WHODAS 2.0 with pain NRS and the Oswestry Disability Index (the NDI is a modification of the later) [19,20]. One third of all the estimated correlations between the Oswestry Disability Index and the WHODAS 2.0 have demonstrated strong correlations while items defining physical functioning seem to correlate more strongly than items defining social or psychological functioning.

Previous studies on the correlation between the WHODAS 2.0 and pain NRS have demonstrated indecisive results [3,5,8,10,21]. In line with the present results, mostly only weak associations have been observed between the WHODAS 2.0 and pain NRS among patients with chronic musculoskeletal pain [20]. For low back pain, correlations between pain and functioning in longitudinal studies seemed to correlate more strongly than in cross-sectional studies [21].

In this study, the NDI items ‘sleeping’, ‘headaches’, ‘reading’ and ‘pain intensity’ did not strongly correlate with any of the single items of the WHODAS 2.0. Neck pain has often been associated with disability and some of the items of the WHODAS 2.0 defining physical functioning could be expected to have stronger correlations with ‘pain intensity’ than they had in the present study [22,23]. On the other hand, even more often, previous studies have observed only loose association between pain severity and disability level [24–27]. Among patients with osteoarthritis, pain itself has not been predictive for disability, but rather obesity, older age, and living in urban area [27]. This dissociation between pain severity and disability might be explained through the ICF framework – while pain is a symptom of physical damage of the body, disability is a much wider sum of many biopsychosocial factors that affect the perceived limitation of functioning. Age-related dependency of perceiving pain and disability might play some role here – a relationship between pain intensity and disability has been shown to be weaker among older people [24]. Additionally, such generic scale as the WHODAS includes broad questions on quality of life and participation, which might distort the correlation between pain and disability as well [26]. One previous study has not found strong correlations among disability, pain and physical impairments among people with neck pain concluding that “clinicians should address as many relevant aspects of a presenting clinical entity as possible in the management of neck pain” [28].

Nevertheless, the exact reasons for the weak correlation between disability and pain remain largely unknown and they might be an important topic for further investigation. Also, future research on age-related differences in this correlation might be important.

## Conclusions

Most of the items and the composite scores of the WHODAS and the NDI demonstrated significant positive correlations. Pain intensity, as defined by the NDI, did not correlate with disability severity measured by the WHODAS 2.0. Also, the NDI items ‘headaches’ and ‘sleeping’ were associated with the WHODAS 2.0 only loosely. It seems that one of these two scales may not directly be substituted by another. When used simultaneously, The WHODAS and the NDI may complement each other covering comprehensively the different dimensions of functioning among people with neck pain.

## Supporting information

S1 Table Spearman´s rank correlations between the 12-item WHO Disability Assessment Schedule 2.0 and the Neck Disability Index. Dancy & Reidy gradation of correlation strength. All the p-values < 0.0001.
(DOCX)

S2 Table Correlations between WHODAS 2.0 and NDI items by sex. (DOCX)
