# Supplementary material for: Convergent validity of 12-item World Health Organization Disability Assessment Schedule (WHODAS 2.0) among people with neck pain
Source: PLoS One. 2025 Mar 11;20(3):e0315676. doi: 10.1371/journal.pone.0315676 (PMC11896042; doi:10.1371/journal.pone.0315676)
Supplement: S1 Table — Dancy & Reidy gradation of correlation strength. All the p-values < 0.0001. (DOCX) [file pone.0315676.s001.docx]

S1 Table. Spearman´s rank correlations between the 12-item WHO Disability Assessment Schedule 2.0 and the Neck Disability Index. Dancy & Reidy gradation of correlation strength. All the p-values <0.0001.

| Neck Disability Index items | WHODAS 2.0 items | | | | | | | | | | | | |
| --- | --- | --- | --- | --- | --- | --- | --- | --- | --- | --- | --- | --- | --- |
|  | Standing | Household care | Learning new task | Community activities | Emotional affection | Concentrating | Walking | Washing | Dressing | Dealing with strangers | Maintaining friendship | Work or school | Total |
| Pain intensity | 0.16 | 0.23 | 0.13 | 0.21 | 0.19 | 0.21 | 0.17 | 0.21 | 0.16 | 0.13 | 0.18 | 0.25 | 0.25 |
| Personal care | 0.32 | 0.42 | 0.2 | 0.36 | 0.25 | 0.29 | 0.35 | 0.56 | 0.53 | 0.18 | 0.23 | 0.35 | 0.47 |
| Lifting | 0.38 | 0.48 | 0.26 | 0.41 | 0.26 | 0.28 | 0.41 | 0.41 | 0.32 | 0.21 | 0.29 | 0.45 | 0.49 |
| Reading | 0.32 | 0.36 | 0.25 | 0.36 | 0.27 | 0.34 | 0.31 | 0.3 | 0.23 | 0.25 | 0.3 | 0.36 | 0.42 |
| Headaches | 0.19 | 0.23 | 0.11 | 0.29 | 0.21 | 0.24 | 0.17 | 0.18 | 0.15 | 0.17 | 0.23 | 0.24 | 0.27 |
| Concentration | 0.39 | 0.38 | 0.46 | 0.49 | 0.47 | 0.64 | 0.38 | 0.37 | 0.25 | 0.43 | 0.49 | 0.41 | 0.59 |
| Work | 0.42 | 0.55 | 0.3 | 0.48 | 0.34 | 0.41 | 0.46 | 0.48 | 0.38 | 0.28 | 0.35 | 0.66 | 0.61 |
| Driving | 0.34 | 0.4 | 0.27 | 0.43 | 0.32 | 0.37 | 0.33 | 0.37 | 0.27 | 0.28 | 0.32 | 0.43 | 0.48 |
| Sleeping | 0.28 | 0.33 | 0.22 | 0.29 | 0.28 | 0.32 | 0.31 | 0.35 | 0.3 | 0.25 | 0.24 | 0.33 | 0.39 |
| Recreation | 0.35 | 0.43 | 0.26 | 0.46 | 0.34 | 0.34 | 0.37 | 0.35 | 0.28 | 0.21 | 0.32 | 0.45 | 0.49 |
| Total | 0.43 | 0.52 | 0.34 | 0.51 | 0.39 | 0.47 | 0.45 | 0.49 | 0.38 | 0.34 | 0.4 | 0.55 | 0.6 |

All the p-values <0.0001

|  | 0.01 to 0.39 weak correlation |
| --- | --- |
|  | 0.40 to 0.69 moderate correlation |
|  | 0.70 to 0.99 strong correlation |
