# Supplementary material for: Convergent validity of 12-item World Health Organization Disability Assessment Schedule (WHODAS 2.0) among people with neck pain
Source: PLoS One. 2025 Mar 11;20(3):e0315676. doi: 10.1371/journal.pone.0315676 (PMC11896042; doi:10.1371/journal.pone.0315676)
Supplement: S2 Table — (DOCX) [file pone.0315676.s002.docx]

S2 Table. Correlations between WHODAS 2.0 and NDI items by sex.

| WHODAS 2.0 →  NDI items ↓ | 1 | 2 | 3 | 4 | 5 | 6 | 7 | 8 | 9 | 10 | 11 | 12 | Total |
| --- | --- | --- | --- | --- | --- | --- | --- | --- | --- | --- | --- | --- | --- |
| Men |  |  |  |  |  |  |  |  |  |  |  |  |  |
| 1 | 0.18 | 0.22 | 0.20 | 0.23 | 0.20 | 0.25 | 0.20 | 0.17 | 0.13 | 0.18 | 0.17 | 0.22 | 0.25 |
| 2 | 0.34 | 0.44 | 0.21 | 0.37 | 0.26 | 0.27 | 0.39 | 0.46 | 0.49 | 0.19 | 0.22 | 0.30 | 0.43 |
| 3 | 0.37 | 0.40 | 0.25 | 0.38 | 0.25 | 0.29 | 0.42 | 0.37 | 0.29 | 0.24 | 0.26 | 0.37 | 0.45 |
| 4 | 0.35 | 0.38 | 0.27 | 0.33 | 0.26 | 0.37 | 0.35 | 0.29 | 0.27 | 0.28 | 0.25 | 0.31 | 0.41 |
| 5 | 0.23 | 0.25 | 0.13 | 0.30 | 0.16 | 0.27 | 0.30 | 0.15 | 0.16 | 0.16 | 0.19 | 0.23 | 0.28 |
| 6 | 0.42 | 0.37 | 0.45 | 0.44 | 0.42 | 0.68 | 0.37 | 0.26 | 0.23 | 0.43 | 0.45 | 0.33 | 0.53 |
| 7 | 0.45 | 0.53 | 0.30 | 0.44 | 0.36 | 0.40 | 0.50 | 0.44 | 0.37 | 0.24 | 0.32 | 0.60 | 0.59 |
| 8 | 0.36 | 0.41 | 0.24 | 0.37 | 0.28 | 0.36 | 0.31 | 0.35 | 0.28 | 0.25 | 0.25 | 0.40 | 0.45 |
| 9 | 0.35 | 0.35 | 0.28 | 0.31 | 0.33 | 0.39 | 0.35 | 0.36 | 0.36 | 0.27 | 0.26 | 0.39 | 0.42 |
| 10 | 0.40 | 0.45 | 0.26 | 0.40 | 0.36 | 0.39 | 0.37 | 0.32 | 0.27 | 0.19 | 0.27 | 0.45 | 0.47 |
| Total | 0.45 | 0.51 | 0.35 | 0.48 | 0.38 | 0.49 | 0.48 | 0.45 | 0.40 | 0.34 | 0.35 | 0.50 | 0.58 |
| Women |  |  |  |  |  |  |  |  |  |  |  |  |  |
| 1 | 0.16 | 0.24 | 0.10 | 0.20 | 0.19 | 0.20 | 0.16 | 0.23 | 0.17 | 0.11 | 0.18 | 0.27 | 0.25 |
| 2 | 0.31 | 0.42 | 0.20 | 0.36 | 0.25 | 0.30 | 0.33 | 0.60 | 0.54 | 0.17 | 0.24 | 0.38 | 0.49 |
| 3 | 0.39 | 0.52 | 0.27 | 0.43 | 0.26 | 0.28 | 0.41 | 0.44 | 0.34 | 0.20 | 0.30 | 0.48 | 0.52 |
| 4 | 0.31 | 0.35 | 0.23 | 0.37 | 0.27 | 0.34 | 0.29 | 0.30 | 0.22 | 0.24 | 0.33 | 0.39 | 0.43 |
| 5 | 0.18 | 0.21 | 0.11 | 0.29 | 0.24 | 0.25 | 0.12 | 0.21 | 0.14 | 0.20 | 0.25 | 0.24 | 0.27 |
| 6 | 0.37 | 0.39 | 0.47 | 0.51 | 0.50 | 0.62 | 0.39 | 0.42 | 0.25 | 0.43 | 0.51 | 0.45 | 0.61 |
| 7 | 0.40 | 0.57 | 0.30 | 0.50 | 0.33 | 0.42 | 0.43 | 0.49 | 0.38 | 0.31 | 0.36 | 0.68 | 0.62 |
| 8 | 0.33 | 0.39 | 0.28 | 0.45 | 0.34 | 0.38 | 0.34 | 0.37 | 0.27 | 0.30 | 0.36 | 0.44 | 0.50 |
| 9 | 0.24 | 0.32 | 0.19 | 0.29 | 0.25 | 0.29 | 0.29 | 0.34 | 0.26 | 0.25 | 0.23 | 0.30 | 0.38 |
| 10 | 0.32 | 0.42 | 0.27 | 0.49 | 0.32 | 0.32 | 0.37 | 0.36 | 0.29 | 0.21 | 0.34 | 0.45 | 0.49 |
| Total | 0.42 | 0.53 | 0.34 | 0.53 | 0.40 | 0.48 | 0.44 | 0.51 | 0.37 | 0.35 | 0.43 | 0.57 | 0.61 |

All the p-values <0.0001

|  | 0.01 to 0.19 negligible correlation |
| --- | --- |
|  | 0.20 to 0.29 weak correlation |
|  | 0.30 to 0.39 moderate correlation |
|  | 0.40 to 0.69 strong correlation |
